# Complications and long-term outcomes after endovascular treatment of basilar trunk aneurysms

**DOI:** 10.3389/fneur.2025.1628676

**Published:** 2025-08-29

**Authors:** Yuange Bi, Yangyang Tian, Yunbo Chi, Xiaohan Chen, Xiaopeng Song, Xuan Chen, Zhongxi Yang, Jing Zhou

**Affiliations:** ^1^Department of Neurosurgery, First Hospital of Jilin University, Changchun, China; ^2^Department of Radiotherapy, Third Hospital of Jilin University, Changchun, China; ^3^School of Nursing, Jilin University, Changchun, China

**Keywords:** intracranial aneurysm, basilar aneurysms, endovascular treatment, basilar trunk artery, complication

## Abstract

**Purpose:**

Basilar trunk aneurysms (BTAs) are rare and challenging to treat, with a high complication rate. This study aimed to analyze and evaluate the complications and long-term outcomes associated with endovascular treatment (EVT) of BTAs and identify risk factors for post-procedural complications and unfavorable clinical outcomes.

**Methods:**

This retrospective, observational cohort study included 90 patients (92 BTAs) treated with EVTs from January 2011 to April 2023. Factors associated with post-procedural complications and unfavorable clinical outcomes were analyzed.

**Results:**

All 90 patients (92 aneurysms) were successfully treated, of which 63 (68.5%) aneurysms were small, 26 (28.3%) were large, and 3 (3.3%) were giant; 23 (25%) were saccular, and 69 (75%) were fusiform and/or dissecting. A total of 36 (40.0%) patients were admitted with ruptured aneurysm. The median follow-up duration was 51.0 months (IQR 21.0–3.0). The favorable clinical outcome rate was 75.6% (68/90). The overall complication rate was 33.33% (30/90), with ischemic and hemorrhagic complications occurring in 25.6% (23/90) and 2.2% (2/90) of cases, respectively. The mortality rate was 8.9% (8/90). Unilateral vertebral artery sacrifice (*P* = 0.015), Glasgow Coma Scale (GCS) grade ≤12 before the procedure (*P* < 0.047), and age ≥60 years (*P* = 0.006) were associated with overall post-procedural complications. Diabetes mellitus (*P* = 0.002), ischemic onset (*P* = 0.005), aneurysms involving the vertebrobasilar junction (*P* = 0.025), and GCS grade ≤12 before the procedure (*P* < 0.001) were risk factors for unfavorable clinical outcomes. Cumulative survival rates at 1, 3, and 5 years were 95.4%, 92.1%, and 87.9%, respectively. The cumulative complication-free survival rates were 72.1%, 68.3%, and 64.5%, respectively. Angiographic follow-up revealed complete occlusion in 71.7% (33/46).

**Conclusion:**

EVT for BTAs appears feasible and yields favorable clinical outcomes at the last follow-up, with reasonable cumulative survival rates at 1, 3, and 5 years. However, clinicians should be vigilant for procedure-related complications, particularly ischemic complications, which may lead to poor outcomes or death.

## Introduction

Basilar trunk aneurysms (BTAs) are uncommon, comprising ~2% of all intracranial aneurysms and 8% of posterior circulation aneurysms (1–3). Their treatment presents challenges primarily due to factors such as deep location, difficult exposure, and characteristics like wide necks, large/giant sizes, or fusiform dilation (2–6). Microsurgical intervention often results in suboptimal outcomes, while endovascular treatment (EVT) is considered the primary approach for BTAs (7). Flow diversion (FD), a subtype of EVT, has gained popularity in managing posterior circulation aneurysms (8–10). However, this technique still presents a relatively high incidence of post-procedural complications and mortality. The literature on EVT outcomes for BTAs remains limited, highlighting the need for further investigation into long-term clinical outcomes and the identification of risk factors for complications leading to disability or death. This study aims to analyze the complications, long-term outcomes, and predictive factors associated with the endovascular management of BTAs.

## Materials and methods

### Study design

This single-center retrospective, observational cohort study examined a database of patients with BTAs who underwent EVTs for these aneurysms at a specific hospital between January 2011 and April 2023. The study was conducted in accordance with the Declaration of Helsinki (as revised in 2013). The institutional review board of the First Hospital of Jilin University approved the study, and individual consent for this retrospective analysis was waived.

### Participants

The decision regarding aneurysm management and the optimal treatment approach was collaboratively evaluated by a multidisciplinary team comprising interventional neuroradiologists and vascular neurosurgeons, considering the lesion's anatomical position, morphological characteristics, and its spatial relationship with the parent vessel. Patients received comprehensive counseling about all available therapeutic options, encompassing cost considerations, insurance reimbursement policies, anticipated post-operative hospitalization duration, procedural trauma, potential serious post-procedural adverse events and variations in post-operative medication regimens for each technique. Subsequently, through personalized consultations with their treating physicians, patients made informed selections between endovascular intervention or other treatment method, including open surgery or conservative treatment.

#### Inclusion criteria

(1) BTAs located between the vertebrobasilar junction (VBJ) and the origin of the superior cerebellar artery; (2) BTAs diagnosed through digital subtraction angiography (DSA); (3) aneurysms originated entirely from BAT or aneurysms originated from BAT but partially involved the VBJ; (4) non-traumatic or iatrogenic origin of the aneurysm; (5) treatment with EVTs; and (6) availability of clinical follow-up data.

#### Exclusion criteria

**Figure 1:**
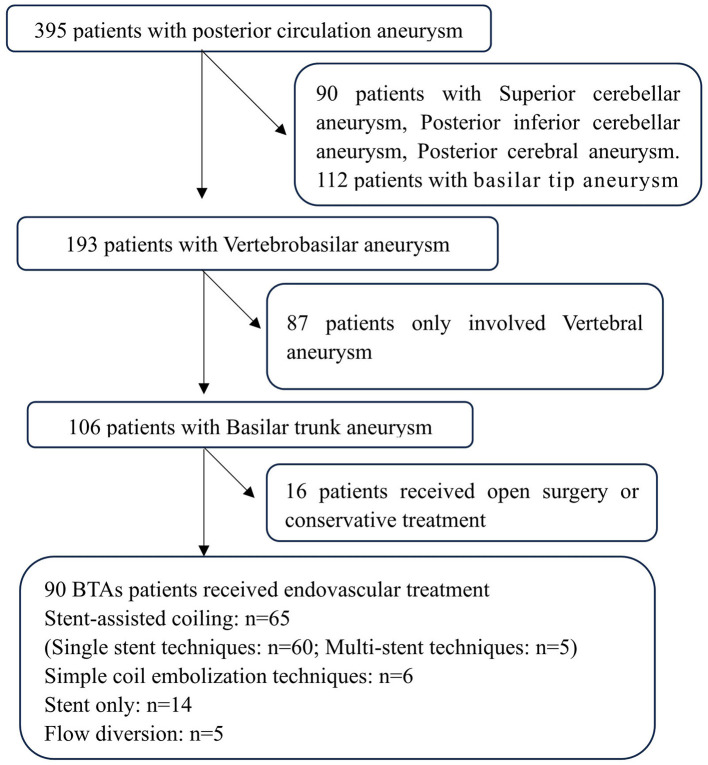
Flowchart of the patient selection process.

(1) Basilar tip aneurysm, superior cerebellar artery aneurysm, posterior cerebral artery aneurysm, or aneurysms on branch vessels of the basilar artery; (2) complications arising from other treated cerebral aneurysms; and (3) Patients with an unfavorable condition prior to the procedure (Modified Rankin Scale (mRS) > 2). The flow chart was shown in Figure 1.

### Antiplatelet therapy

Before the endovascular procedure for ruptured aneurysms, either tirofiban or a loading dose of 300 mg clopidogrel and 300 mg aspirin was administered. All ruptured aneurysms received treatment within 24 h of admission. For unruptured aneurysms, oral administration of clopidogrel (75 mg/day) and aspirin (100 mg/day) was initiated at least 3–5 days before the procedure. Following the administration of dual oral antiplatelet agents, platelet function was monitored in all patients using thromboelastography (TEG). If the results did not reach the desired range, clopidogrel (75 mg/day) was switched to ticagrelor (90 mg twice daily). Post-procedure, clopidogrel (75 mg/day) was continued for a minimum of 3 months (6 months for patients treated with flow diversion), while aspirin (100 mg/day) was maintained indefinitely.

### Procedure

Neurointerventionalists, each with over 10 years of experience in neurointerventional surgery, determined the EVT modalities based on aneurysm anatomical factors. All EVTs were performed under general anesthesia. Stent-assisted coiling utilized conventional stents, including the Low-profile Visualized Intraluminal Support (LVIS) device (MicroVention Terumo, USA), Enterprise (Cordis Neurovascular, USA), self-expandable intracranial stent Neuroform (Stryker Neurovascular, USA), LEO (Balt, Extrusion, Montmorency, France), and Solitaire (ev3 neurovascular, Irvine, CA, USA) stents. Coils were deployed until satisfactory aneurysm occlusion was achieved, or when additional packing was not feasible. Flow diverters (FD) included the Tubridge (MicroPort NeuroTech, China) and Pipeline (Medtronic, USA) devices. Post-procedural multiangle unsubtracted angiography and VasoCT were used to assess FD expansion. In cases of inadequate FD expansion, balloon angioplasty or in-stent massage with a microcatheter and microguidewire was performed.

### Date collocation

Baseline characteristics of patients, including sex, age, hypotension, diabetes mellitus, smoking, and alcohol abuse, were obtained. Aneurysm baseline characteristics, such as location, size, morphology, and involvement of branch vessels, were also documented. Procedural details and complications were extracted from medical records. Imaging examinations were performed to determine aneurysm number, size, shape, and parent artery features. The initial onset was categorized as ischemic symptoms, hemorrhage, or other presentations, such as headache, mass effect, and incidental findings.

Post-procedural complications, including rebleeding, ischemia, shunt-dependent hydrocephalus, or other threatening events leading to hospitalization or prolonged hospitalizations, were recorded. Ischemic events were defined as: (1) the onset of new neurological deficits in the post-procedural period, in-stent thrombosis, or partial or complete occlusion of the proximal or distal arteries on digital subtraction angiography (DSA), and (2) the onset of new neurological deficits in the post-procedural period and/or thromboembolic symptoms (excluding vasospasm) with or without corresponding cerebral infarction on magnetic resonance imaging (MRI) or CT. Hemorrhagic events were defined as: (1) post-procedural CT/MRI showing new intracerebral hemorrhage with or without clinical symptoms and (2) new subarachnoid hemorrhage on CT. Endovascular procedure-related data, complications, and patient neurological status were retrospectively collected and analyzed.

### Follow-up

Clinical outcome data were collected through neurological examinations or telephone calls at 30 days, 6 months, and during annual follow-ups. The mRS score was used for clinical follow-up, with an mRS score ≤ 2 considered a favorable outcome. Angiographic follow-up with DSA was performed at 6 months and 1–2 years post-procedure to assess aneurysm occlusion status. Angiographic outcomes were evaluated using the Raymond-Roy grading scale, where Grade I indicated complete aneurysm occlusion, Grade II signified opacification of the aneurysm neck, and Grade III denoted opacification between the coil mass and aneurysm wall.

### Statistical analysis

Qualitative variables were expressed as numbers and percentages. Continuous variables with a normal distribution were presented as mean ± SD, while continuous variables with a non-normal distribution were represented by the median and interquartile range (IQR).

Analysis of variables between the two groups was carried out by using the Mann–Whitney *U*-test or independent sample *t*-test for continuous variables and the chi-squared test or the Fisher's exact test for qualitative variables. Kaplan-Meier survival analysis was used to calculate the cumulative survival rate during follow-up. Univariate and multivariate Cox regression analyses were performed to identify risk factors for overall treatment-related complications and unfavorable clinical outcomes. Variables identified as potential predictors in the univariate analysis (*P* < 0.1) were included in the multivariate Cox regression analyses. Cases with missing follow-up data were excluded. A *P*-value of < 0.05 was considered statistically significant. Statistical analyses were performed using SPSS software version 26 (IBM Corp., Armonk, NY, USA).

## Results

### Patient and aneurysm characteristics

This study included 90 patients (92 aneurysms) with a mean age of 54.5 ± 10.5 years (range, 22–74) (Figure 1). The cohort consisted of 60 male patients (66.7%) and 30 female patients (33.3%). Of the aneurysms, 23 (25%) were saccular, and 69 (75%) were fusiform and/or dissecting. Seventeen aneurysms (18.5%) involved the vertebrobasilar junction (VBJ). Six (6.7%) patients had vertebrobasilar dolichoectasia. The median maximum diameter of the aneurysms was 7.19 mm (IQR, 4.8–10.68). For the 36 patients (40%) with ruptured aneurysms, 26 were classified as Hunt–Hess grade I or II (72.2%) and 10 as grade III–V (27.7%). Regarding the Glasgow Coma Scale (GCS) before the procedure, 79 patients had a GCS grade >12 (87.8%) and 11 had a GCS grade ≤12 (12.2%). Among the 90 patients, 36 presented with a hemorrhagic event (40.0%), 27 with ischemic symptoms (32.2%), and 27 with no symptoms (27.8%) (Table 1).

**Table 1:** Baseline characteristics and outcomes.

**Variables**	***n* = 90 patients (92 aneurysms)**
**Age (years)**, ***n*** **(%)**	
< 60	58 (64.4)
≥60	32 (35.6)
Female, *n* (%)	30 (33.3)
Hypertension, *n* (%)	48 (53.3)
Diabetes mellitus, *n* (%)	12 (13.3)
Smoking, *n* (%)	46 (51.1)
Alcohol abuse, *n* (%)	31 (34.4)
**Onset symptoms**, ***n*** **(%)**	
Ischemic stroke	29 (32.2)
Hemorrhagic event	36 (40.0)
Others^†^	25 (27.8)
**HH before procedure**, ***n*** **(%)**^‡^	
≤ 2	26 (72.2)
>2	10 (27.8)
**mRS grade before procedure**, ***n*** **(%)**	
0	32 (35.6)
1	49 (54.4)
2	9 (10.0)
**GCS grade before procedure**, ***n*** **(%)**	
>12	79 (87.8)
≤ 12	11 (12.2)
Patients with vertebrobasilar dolichoectasia	6 (6.7)
**Aneurysm size**, ***n*** **(%)**^§^	
< 10 mm	63 (68.5)
10–25 mm	26 (28.3)
≥25 mm	3 (3.3)
**Aneurysm morphology**	
Fusiform/dissecting	69 (75.0)
Saccular	23 (25.0)
Aneurysm involved vertebrobasilar junction, *n* (%)	17 (18.5)
Procedure time, min (mean ± SD)	135.6 ± 43.6
Unilateral vertebral artery sacrifice, *n* (%)	2 (2.2)
**Type of stents**, ***n*** **(%)**	
Enterprise	30 (33.3)
PED	4 (4.4)
Tubridge	1 (1.1)
LVIS	16 (17.8)
Enterprise +LEO	1 (1.1)
Enterprise + LVIS	1 (1.1)
LVIS+ solitaire	1 (1.1)
Solitaire	21 (23.3)
Neuroform	8 (8.9)
LEO	1 (1.1)
Overall complications, *n* (%)	30 (33.3)
Ischemia	23 (25.6)
Hemorrhage	2 (2.2)
Others	1 (1.1)
Death	8 (8.9)
**Periprocedural complications**, ***n*** **(%)**^||^	
Ischemia	12 (13.3)
Hemorrhage	1 (1.1)
Shunt-dependent hydrocephalus	4 (4.4)
Death	1 (1.1)
**Follow-up complication**, ***n*** **(%)**	
Ischemia	11 (12.2)
Hemorrhage	1 (1.1)
Others^††^	1 (1.1)
Death	7 (7.8)
**mRS at discharge**, ***n*** **(%)**	
≤ 2	78 (86.7)
>2	12 (13.3)
**mRS at last follow-up**, ***n*** **(%)**	
0–2	68 (75.6)
3–6	22 (24.4)
**Follow-up angiographic outcome**, ***n*** **(%)**^‡‡^	
Completely occluded	33 (71.7)
Incompletely occluded	13 (28.3)

mRS, modified Rankin Scale; PED, pipeline embolization device; LVIS, Low-profile Visualized Intraluminal Support; GCS, Glasgow Coma Scale; HH, Hunt-Hess Scale.

^†^Others include headache, mass effect, and incidental.

^‡^*n* = 36.

^§^*n* = 92.

^||^1 patient had ischemic events during the periprocedural period and hemorrhagic events in the follow-up, while 2 patients had ischemic events during the periprocedural period and did not survive the follow-up.

^††^Others included heart failure.

^‡‡^*n* = 46.

### Procedural details

The technical success rate was 100% for all 90 patients. Stent-assisted coiling embolization was performed in 65 patients (72.2%), including 60 patients (66.7%) who underwent single stent-assisted coiling embolization and 5 patients (5.6%) who underwent multi-stent-assisted coiling embolization. Simple coil embolization was performed in 6 patients (6.7%), while 14 patients (15.6%) underwent stent placement without coiling. Flow diversion (FD) was used in 5 patients (5.6%). The median procedure time per patient was 130 min (IQR, 111.5–151.3), and the median coil count was 4 (IQR, 2–5.25) (Table 1).

### Clinical outcomes

Complications occurred in 30 patients (33.3%), including 23 patients (25.6%) with ischemic events, 2 patients (2.2%) with hemorrhagic events, 4 patients (4.4%) with shunt-dependent hydrocephalus, and 1 patient (1.1%) with heart failure. Sixteen patients (17.8%) experienced complications during the periprocedural period, while 14 patients (15.6%) had complications during follow-up. One patient had ischemic events during the periprocedural period and hemorrhagic events during follow-up, while two patients had ischemic events during the periprocedural period and died during follow-up. At discharge, 78 patients (86.7%) had mRS scores of 0–2, while 6 patients (6.7%) were dependent or deceased (mRS score of 3–6). Clinical follow-up was conducted for all survivors, with a median follow-up duration of 51.0 months (IQR 21.0–73.0). At the last follow-up, outcomes were favorable (mRS score of 0–2) in 68 cases (75.6%), and the overall mortality rate was 8.9% (8/90). Detailed outcomes are presented in Table 1.

### Predictive factors for overall complications

In the univariate regression analysis, age ≥60 years (*P* = 0.022), aneurysm involvement of the VBJ (*P* = 0.074), ischemic onset (*P* = 0.087), unilateral vertebral artery sacrifice (*P* = 0.015), and GCS grade ≤ 12 before the procedure (*P* = 0.040) were associated with post-procedural complications. In the multivariate Cox regression analysis, statistically significant factors associated with overall post-procedural complications included unilateral vertebral artery sacrifice (HR: 7.37, 95% CI: 1.48–37.03; *P* = 0.015), age ≥60 years (HR: 2.92, 95% CI: 1.36–6.26; *P* = 0.006), and GCS grade ≤ 12 before the procedure (HR: 2.50, 95% CI: 1.01–6.19; *P* = 0.047). A summary of the univariate and multivariate Cox regression analysis for overall post-procedural complications is presented in Table 2.

**Table 2:** Univariate and multivariate analyses of factors impacting post-procedural complications.

**Parameter**	**Univariable analysis**			**Multivariable analysis**		
	**HR**	**95%CI**	***P*** **-value**	**HR**	**95%CI**	***P*** **-value**
Age ≥ 60	2.34	1.13–4.83	0.022	2.92	1.36–6.26	0.006^**^
Sex (male)	0.58	0.28–1.20	0.144	–	–	–
Smoking	0.76	0.37–1.56	0.453	–	–	–
Drinking	0.86	0.40–1.84	0.692	–	–	–
Hypertension	1.62	0.77–3.40	0.206	–	–	–
Diabetes	1.82	0.74–4.47	0.190	–	–	–
Ruptured	0.83	0.39–1.75	0.621	–	–	–
Ischemic onset	1.93	0.91–4.09	0.087	1.55	0.72–3.34	0.265
Aneurysm involved VBJ	1.95	0.94–4.07	0.074	1.44	0.66–3.14	0.360
Aneurysm size (≥25 mm)	1.26	0.70–2.27	0.448	–	–	–
Patients with VBD	0.46	0.06–3.34	0.439	–	–	–
Morphology (fusiform/dissecting)	0.87	0.37–2.02	0.744	–	–	–
Stenosis of the parent arteries	0.70	0.17–2.93	0.622	–	–	–
Braided stent	0.62	0.24–1.62	0.331	–	–	–
Engrave stent	1.23	0.56–2.68	0.609	–	–	–
Multi-stent	0.72	0.25–2.06	0.535	–	–	–
Unilateral vertebral artery sacrifice	6.30	1.43–27.69	0.015	7.37	1.48–37.03	0.015^*^
GCS grade ≤ 12	2.43	1.04–5.69	0.040	2.50	1.01–6.19	0.047^*^
HH grade ≥3	1.32	0.46–3.79	0.612	–	–	–
Procedure time (min)	1.00	1.00–1.01	0.291	–	–	–
Stent used	0.55	0.17–1.83	0.330	–	–	–

VBJ, vertebrobasilar junction; VBD, vertebrobasilar dolichoectasia; GCS, Glasgow Coma Scale; HH, Hunt-Hess Scale. ^*^p < 0.05; ^**^p < 0.01.

### Predictive factors for unfavorable clinical outcomes

In the univariate Cox regression analysis, diabetes mellitus (*P* = 0.059), ischemic onset (*P* = 0.007), aneurysm involvement of the VBJ (*P* = 0.003), age ≥60 years (*P* = 0.024), and GCS grade ≤ 12 before the procedure (*P* = 0.002) were associated with unfavorable clinical outcomes. In the multivariate Cox regression analysis, diabetes mellitus (HR: 5.24, 95% CI: 1.81–15.12; *P* = 0.002), ischemic onset (HR: 3.56, 95% CI: 1.46–8.70; *P* = 0.005), aneurysm involvement of the VBJ (HR: 2.81, 95% CI: 1.14–6.91; *P* = 0.025), and GCS grade ≤ 12 before the procedure (HR: 15.57, 95% CI: 5.01–48.42; *P* < 0.001) were identified as independent risk factors for unfavorable clinical outcomes (Table 3).

**Table 3:** Univariate and multivariate analyses of factors influencing unfavorable clinical outcomes.

**Parameter**	**Univariable analysis**			**Multivariable analysis**		
	**HR**	**95%CI**	***P*** **-value**	**HR**	**95%CI**	***P*** **-value**
Age ≥ 60	2.66	1.14–6.24	0.024	2.31	0.93–5.75	0.073
Sex (male)	0.54	0.23–1.25	0.152	–	–	–
Smoking	0.50	0.21–1.20	0.119	–	–	–
Drinking	0.69	0.28–1.71	0.417	–	–	–
Hypertension	1.66	0.70–3.97	0.252	–	–	–
Diabetes mellitus	2.47	0.97–6.33	0.059	5.24	1.81–15.12	0.002^**^
Ruptured	0.77	0.32–1.86	0.562	–	–	–
Ischemic onset	3.27	1.38–7.73	0.007	3.56	1.46–8.70	0.005^**^
Aneurysm involved VBJ	3.76	1.58–8.93	0.003	2.81	1.14–6.91	0.025^*^
Aneurysm size (≥25 mm)	1.18	0.58–2.38	0.647	–	–	–
Patients with VBD	0.63	0.09–4.72	0.655	–	–	–
Morphology (fusiform/dissecting)	0.63	0.21–1.85	0.396	–	–	–
Stenosis of the parent arteries	1.20	0.279–5.16	0.807	–	–	–
Braided stent	0.33	0.08–1.42	0.136	–	–	–
Engrave stent	1.40	0.54–3.59	0.487	–	–	–
Multi-stent	1.13	0.38–3.37	0.823	–	–	–
Unilateral vertebral artery sacrifice	4.68	0.60–36.39	0.141	–	–	–
GCS grade ≤ 12	4.22	1.69–10.50	0.002	15.57	5.01–48.42	< 0.001^***^
HH grade ≥3	1.98	0.65–5.75	0.240	–	–	–
Procedure time (min)	1.00	1.00–1.01	0.296	–	–	–
Stent used	0.50	0.15–1.7	0.274	–	–	–

VBJ, vertebrobasilar junction; VBD, vertebrobasilar dolichoectasia; GCS, Glasgow Coma Scale; HH, Hunt-Hess Scale. ^*^p < 0.05; ^**^p < 0.01; ^***^p < 0.001.

### Survival analysis

The overall cumulative survival rates at 1, 3, and 5 years were 95.4%, 92.1%, and 87.9%, respectively. The complication-free cumulative survival rates at 1, 3, and 5 years were 72.1%, 68.3%, and 64.5%, respectively.

### Angiographic outcomes

**Figure 2:**
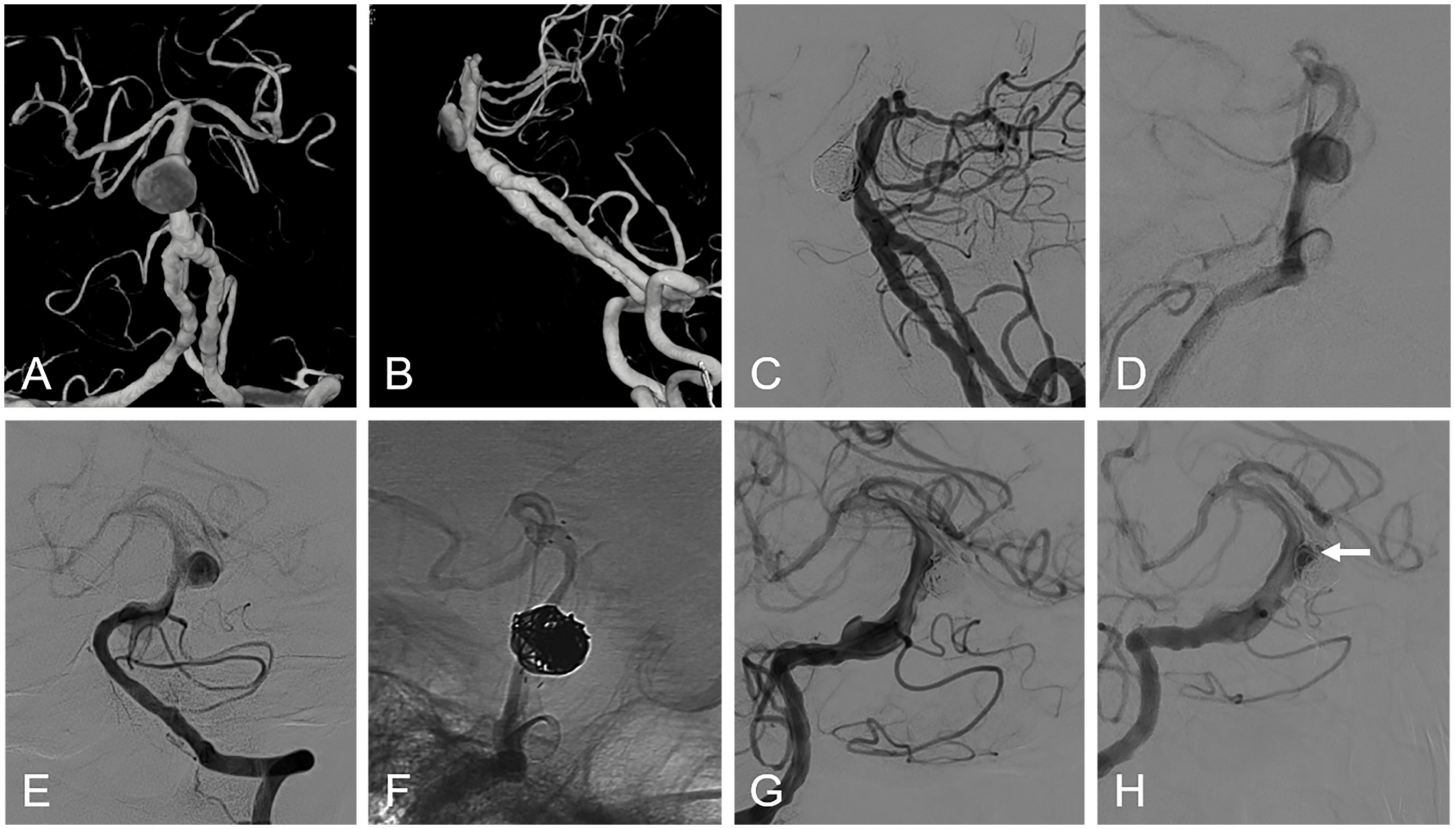
Illustrations of patients with large aneurysms in the middle basilar artery. **(A, B)** display a conventional angiography revealing an aneurysm in the basilar artery trunk. **(C)** shows the patient's treatment using stent-assisted coil embolization. **(D, E)** show the lateral and anteroposterior views of the basilar artery aneurysm. The patient was treated using stent-assisted coil embolization **(F)**. **(G)** exhibits the immediate angiographic results following coil embolization. **(H)**, taken at 6 months, displays angiography indicating partial compression of the coil within the aneurysm lumen, with a small amount of contrast filling in the aneurysm's neck (arrow). The parent vessel and distal branch vessels remain patent, and the patient remains symptom-free.

Angiographic outcomes were available for 46 patients at a median follow-up time of 8.0 months (IQR 6.0–13.0). The overall complete occlusion rate was 71.7% (33/46) (grade I of the Raymond–Roy grading scale), with 40 aneurysms (87.0%) showing adequate occlusion (grades I and II of the Raymond–Roy grading scale) (Figure 2). Two aneurysms (4.3%) were recanalized and subsequently underwent retreatment, while two aneurysms (4.3%) enlarged compared to their original size. One patient (2.2%) experienced unilateral limb weakness 9 months after the procedure, and subsequent DSA indicated occlusion of the parent artery. After intensive antiplatelet therapy, the patient's symptoms were relieved without the need for surgical intervention. Aneurysms involving the vertebrobasilar junction demonstrated significantly lower complete occlusion rates at follow-up compared to those solely affecting the basilar artery trunk (*P* = 0.023) (Supplementary Table S1).

## Discussion

In our study, a total of 90 patients (92 aneurysms) were enrolled. Complications were observed in 30 patients (33.3%), favorable clinical outcomes were achieved in 68 cases (75.6%), and the overall mortality rate was 8.9% (8/90). Factors such as unilateral vertebral artery sacrifice, GCS grade ≤ 12, and age ≥60 years were associated with overall post-procedural complications. Diabetes mellitus, ischemic onset, aneurysms involving the VBJ, and GCS grade ≤ 12 were linked to unfavorable clinical outcomes during follow-up.

The treatment of BTAs poses significant challenges and is consistently associated with high morbidity and mortality rates (8). Surgical interventions for giant or complex BA aneurysms are particularly challenging due to restricted space, intricate anatomy, abundant perforating vessels, and large, irregular shapes, leading to relatively unfavorable clinical outcomes (2, 7). Although endovascular therapy is the primary approach for BTAs, it still carries a relatively high risk of unfavorable outcomes and complications (8–10). Compared to anterior circulation aneurysms, posterior circulation aneurysms are more prone to rupture due to their size, resulting in higher treatment risks regardless of the technique employed (3). Among posterior circulation aneurysms, vertebrobasilar artery aneurysms (VBA) have a poor prognosis, with an elevated risk of growth and rupture, contributing to an overall annual mortality rate of 13% (11). BTAs are associated with a higher incidence of unfavorable clinical outcomes and complications. Studies by Peng et al. (8) and Zhong et al. (10) examined the treatment outcomes of BTAs with EVT. Peng et al. reported a procedure-related complication rate of 23.4% (18/77) in 77 cases treated with EVT. Zhong et al., in their review of 111 patients undergoing reconstructive EVT for BTAs, found periprocedural complication rates of 29.7%, favorable clinical outcomes in 83.8%, and a mortality rate of 14.4%. In our study, we observed similar rates of complications (33.3%), favorable clinical outcomes (75.6%), and mortality (8.9%), aligning with findings from these published studies.

Complications associated with EVT primarily involve hemorrhagic and ischemic events, with a notably high incidence of ischemic complications in cases of posterior circulation aneurysms. In a study by Peng et al. (8), ischemic complications occurred in 13 out of 77 patients (16.9%) with basilar trunk and VBJ aneurysms. Wu et al.'s study reported ischemic complications in 10 out of 34 patients (29.4%) with large BTAs (12). Another study involving 28 patients with BTAs reported an ischemic complication rate of 32.1% (13). In our study, the primary complication observed in BTAs treated with EVT was ischemia (25 out of 90, 27.8%), consistent with findings from previous studies (12, 13). Among these, twelve patients experienced periprocedural ischemic events, and seven patients experienced ischemic events during follow-up. Possible reasons for these ischemic events include inadequate administration of antiplatelet drugs (14), perforator infarction (10), intraluminal thrombus formation (15), or *in-situ* thrombosis within the parent artery (10, 15).

Moreover, hemorrhagic complications are also linked to poor clinical outcomes and higher mortality rates (10, 14). Zhong et al. (10) reported 111 patients with BTAs undergoing reconstructive EVT, with 4 cases (3.6%) experiencing hemorrhagic complications, leading to 2 fatalities. In another study, 8.4% of 424 non-saccular vertebrobasilar aneurysms had hemorrhagic complications, with an overall hemorrhage mortality rate of 6% (16). In our study, the rate of post-procedural subarachnoid hemorrhage was 5.6% (5/90). The mechanism behind post-procedural subarachnoid hemorrhage after EVT remains unclear, but some studies have suggested potential causes, including high-intensity administration of antiplatelet agents (14), a history of hypertension (4, 14, 15), large aneurysm neck diameters (4, 17), flow reversal in distal collaterals (18), vessel damage, and ischemia–reperfusion hemorrhage (12, 15). Furthermore, three patients in our study experienced delayed rupture, with the mechanism remaining unknown. Past studies have proposed that intra-aneurysmal thrombus could trigger increased autolysis, overloading the biological defense mechanisms of the vessel wall and resulting in aneurysm rupture (19). Additionally, increased intra-aneurysmal pressure (20), large/giant saccular aneurysms, and mechanical injury during surgery (19, 20) might collectively contribute to eventual delayed rupture.

We also observed that aneurysms located at the VBJ were associated with unfavorable clinical outcomes. VBJ aneurysms are often linked with basilar artery fenestration (21, 22). The intricate anatomical structure and the protrusion of perforating branches feeding the brainstem and lower cranial nerves from this region make the treatment of VBJ aneurysms challenging (21–23). While studies have confirmed the efficacy and safety of EVT in treating VBJ aneurysms, a high rate of complications and mortality still exists (21, 23). Unilateral vertebral artery sacrifice is one treatment option for VBJ aneurysms, allowing for this approach when blood flow from one vertebral artery directly feeds into the aneurysm lumen while ensuring the patency of the distal posterior inferior cerebellar artery (24). However, the sacrifice of the unilateral vertebral artery is associated with a higher risk of neurologic complications secondary to ischemia (24). Meckel et al. (25) reported 10 patients with VBJ aneurysms who had undergone flow diversion treatment, among whom 9 underwent unilateral vertebral artery sacrifice. During follow-up, 4 of the 10 patients developed ischemic complications and 4 died. In our study, we found that unilateral vertebral artery sacrifice was associated with post-procedural complications. Although the specific mechanism is unclear, some studies suggest that after vertebral artery occlusion, hemodynamic forces are altered in the remaining vessels, which are burdened by an increased cerebral blood flow (23). Thrombus formation in the occluded vertebral artery, which may break off over time, and progressive constriction of the occlusive artery can squeeze the thrombus into collateral arteries, leading to ischemia due to hypoperfusion of collateral arteries or ischemia–reperfusion hemorrhage (12, 23). In addition, previous studies have shown that patients with vertebrobasilar dolichoectasia are more likely to exhibit continued aneurysm growth, and these patients are at higher risk of ischemic stroke and poor outcomes (1, 16, 26). Siddiqui et al. (26) included 7 patients with vertebrobasilar dolichoectasia who had undergone flow diversion treatment, with 4 dying post-procedure. However, our study did not find an association between vertebrobasilar dolichoectasia and post-procedural complications or poor outcomes. This may be related to the limited number of such patients in our case series (only 6 cases), which could also partially explain the favorable outcomes observed in this cohort.

In this study, age ≥60 years was associated with post-procedural complications. Elderly patients often have underlying health conditions, degenerative physical functions, and lower tolerance for surgery (4, 10, 27). Additionally, a history of diabetes mellitus was linked to unfavorable clinical outcomes. This may be due to the fact that diabetes mellitus can increase the risk of artery stenosis, ischemic symptoms, or acute ischemic stroke (28). Patients with diabetes mellitus and acute ischemic stroke tend to experience more complications, worse outcomes, higher mortality rates, and increased stroke recurrence compared to those without diabetes mellitus (28, 29). Furthermore, a pre-procedural GCS score of ≤ 12 was associated with unfavorable clinical outcomes in our study. Neurological and cognitive status at baseline is critical in determining quality of life after aneurysm surgery (30). A GCS score of ≤ 12 indicates severe brain injury and a significant inflammatory and oxidative stress response, which can predict unfavorable outcomes following subarachnoid hemorrhage surgery (31). Additionally, we observed that an ischemic onset was associated with unfavorable clinical outcomes. Ischemic stroke was identified as the most common cause of mortality in cases of vertebrobasilar non-saccular and dolichoectatic aneurysms, with a high ischemia rate of 6% per year (18). This could be attributed to the presence of intra-aneurysmal partial thrombosis, where detachment of the intra-aneurysmal thrombus may lead to occlusion of perforating or distal vessels, resulting in ischemic stroke (15).

Our study has several inherent limitations that need to be acknowledged. Firstly, the small number of patients and the retrospective nature of data collection are significant limitations. The potential bias inherent in retrospective research is unavoidable. Secondly, the diverse shapes and sizes of aneurysms require different treatment methods, and our study spans over 11 years, during which surgical strategies and physician techniques may have evolved, potentially leading to variations in complications and clinical outcomes. Thirdly, angiographic follow-up data for the included patients were limited, and the follow-up times were not uniform, which may have impacted the consistency of aneurysm occlusion observations at regular intervals. In addition, owing to missing data, the influence of the length of the aneurysm-affected segment and the existence of mural thrombus was not assessed in this study. Furthermore, the therapeutic approach of unilateral vertebral artery sacrifice imposed high technical demands and was applicable only to complex cases. This resulted in limited clinical data availability and challenges in generalizing the findings. Finally, our cohort only included 5 cases with flow diversion treatment and lacked experience with modern endovascular options such as intrasaccular flow-disruption devices or newer-generation flow diverters. Consequently, the applicability of these findings may be constrained in settings where such technologies are routinely adopted.

## Conclusion

EVTs for BTAs appear to be generally safe. At 1, 3, and 5 years, the cumulative survival rates were 95.4%, 92.1%, and 87.9%, respectively. Similarly, the complication-free survival rates were 72.1%, 68.3%, and 64.5%, respectively. Factors associated with overall complications after the procedure included age ≥60 years, a pre-procedure GCS grade ≤ 12, and unilateral vertebral artery sacrifice. Unfavorable clinical outcomes during follow-up were linked to diabetes mellitus, ischemic onset, aneurysms involving the VBJ, and a pre-procedure GCS grade ≤ 12. The primary focus of future research should aim to reduce post-procedural complications and improve asymptomatic survival rates.

## Data Availability

The raw data supporting the conclusions of this article will be made available by the authors, without undue reservation.
