# Supplementary material for: Complications and long-term outcomes after endovascular treatment of basilar trunk aneurysms
Source: Front Neurol. 2025 Aug 29;16:1628676. doi: 10.3389/fneur.2025.1628676 (PMC12428029; doi:10.3389/fneur.2025.1628676)
Supplement: Supplementary file 2 [file Table_2.docx]

**Table S1** Aneurysm occlusion status at follow-up

| Parameter | Aneurysm residual  (n=16) | Aneurysm complete occlusion (n=30) | P-value |
| --- | --- | --- | --- |
| Follow-up time(months) | 7.0(6.0, 12.5) | 9.0(6.0, 13.0) | 0.861 |
| Age≥60 | 5(31.3) | 7(23.3) | 0.818 |
| Sex (Male) | 13(81.3) | 19(63.3) | 0.357 |
| Smoking | 7(43.8) | 17(56.7) | 0.404 |
| Drinking | 6(37.5) | 13(43.3) | 0.946 |
| Hypertension | 6(37.5) | 17(56.7) | 0.216 |
| Diabetes mellitus | 0 | 4(13.3) | 0.126 |
| Ruptured | 7(43.8) | 9(30.0) | 0.543 |
| Ischemic onset | 7(43.8) | 17(56.7) | 0.404 |
| Aneurysm involved VBJ | 7(43.8) | 3(10.0) | 0.023 |
| Aneurysm Size(≥10mm) | 5(31.3) | 7(23.3) | 0.818 |
| Morphology (Fusiform/dissecting) | 13(81.3) | 21(70.0) | 0.635 |
| Stenosis of the parent arteries | 1(6.3) | 2(6.7) | 1.000 |
| Braided stent | 4(25.0) | 7(23.3) | 1.000 |
| Engrave stent | 12(75.0) | 21(70.0) | 0.988 |
| Multi-stent | 2(12.5) | 6(20.0) | 0.817 |
| GCS grade≤12 | 1(6.3) | 1(3.3) | 1.000 |
| HH grade≥3 | 1(6.3) | 1(6.3) | 1.000 |
| Stent used | 16(100.0) | 29(96.7) | 1.000 |
